# 1169. Revenge of the Syph(ilis): Investigating Congenital Syphilis at a Tertiary Care Center Amidst a Rising Epidemic

**DOI:** 10.1093/ofid/ofab466.1362

**Published:** 2021-12-04

**Authors:** Tara Ness, Ryan H Rochat, Claire Bocchini, C Mary Healy

**Affiliations:** 1 Baylor College of Medicine/Texas Children’s Hospital, Houston, TX; 2 Texas Children’s Hospital, Baylor College of Medicine, Houston, TX; 3 Baylor College of Medicine, Houston, TX

## Abstract

**Background:**

Congenital syphilis is a chronic infection acquired by the fetus in utero from a mother infected with *Treponema palladium*. It has a large spectrum of disease manifestations from asymptomatic infection to blindness, abnormal bone and teeth formation, deafness, or even death. Despite antenatal screening and the availability of effective treatment the incidence of congenital syphilis has risen since 2012, with 23.3 cases per 100,000 live births in 2017 according to the Centers for Disease Control. We sought to investigate the epidemiology, clinical manifestations, and treatment outcomes of congenital syphilis.

**Methods:**

We undertook a retrospective review of individuals born at Texas Children’s Hospital from January 1, 2010 to May 15, 2021 to evaluate compliance with current diagnostic and treatment recommendations. Diagnostic and billing codes for congenital syphilis were used to generate a list of subjects. Patient demographics and clinical details were abstracted from the electronic medical record (EMR). Statistics were performed using Microsoft Excel.

**Results:**

107 children (52% male, 48% female) were identified from diagnostic and billing codes in the EMR under the SNOMED-CT diagnosis of “congenital syphilis” and were less than two years of age at the time of diagnosis. All received penicillin within one month of diagnosis. 94 of these had a skeletal x-ray performed, with 11 (12%) having an abnormal skeletal x-ray consistent with congenital syphilis. 88 (82%) had a lumbar puncture done with a quantitative CSF VDRL obtained. 88 received aqueous penicillin G for proven/highly probable or possible syphilis. Four patients were deceased at the time of data inquiry. Of those with abnormal skeletal x-rays, “metaphyseal lucency” was the most common finding.

**Conclusion:**

Congenital syphilis remains a significant concern in the United States and carries the risk of significant long-term morbidity for infants and children. Antenatal screening with appropriate treatment in pregnancy and adequate follow-up would decrease the need for neonatal evaluation and treatment.

**Disclosures:**

**C. Mary Healy, MD**, **Dexcom** (Shareholder)**Intuitive** (Shareholder)**Quidel Corporation** (Shareholder)**Up to Date** (Other Financial or Material Support, Honorarium)**Vapotherm** (Shareholder)

